# The Institutional Library

**Published:** 1917-07-07

**Authors:** 


### THE INSTITUTIONAL LIBRARY.

**The Causes of Tuberculosis.** By LOUIS COBBETT, M.D., F.R.C.S., University Lecturer in Pathology, Cambridge. (Cambridge University Press. 1917. Demy 8vo, pp. xiii + 707, with 23 Plates.)

A BETTER title for this book would have been "The Comparative Bacteriology of Tuberculosis." On that subject it gives the student of tuberculosis the last word from the laboratory, and is far and away the best text-book in the language. In other departments, although of course reaching a good standard of performance, there is visible a falling off. More or less second-hand sources are quoted too often in treating of demography and epidemiology. Particularly does the author's inevitable lack of clinical knowledge handicap him. He states that there is "absolutely no reason to think that it [tuberculosis] is correlated with any other weakness," and quotes a provincial medical officer of health's remark that it is absurd, because half the Sheffield grinders die from tuberculosis of the lungs, to say that half the Sheffield grinders are therefore weaklings, adding that he himself has been greatly struck by the fact that among friends and acquaintances attacked by the disease many were physically considerably above the average. Thus do the sanitarian and the pathologist, the one from observation of a peculiarly predisposed class, the other from the narrow circle of a single personal acquaintance, speak without the book of a complete knowledge of tubercle. Is not congenital heart disease a "weakness," and is not its association with phthisis a commonplace of the text-books? Or Mongolian idiocy, 93 per cent. of the subjects of which, living under good conditions at Earlswood, die from tuberculosis? Or lunacy, while the high death-rate from phthisis of lunatics is notorious? Or alcoholism, which "fait le lit de la tuberculose"? Or a condition of underweight, which Parkes Weber and Kirkness, and indeed every medical officer to a life insurance company, describe as predisposing to phthisis? This statement is an egregious error, which should be removed from the next edition. By far the greater part of the work treats of bacteriology, however. Much's granules and the "Splitter" forms are not mentioned, but as an account of the great amount of work done in this country, largely as a result of the appointment of the Royal Commission on Tuberculosis, it is very valuable. Although a text-book, it is far from being dogmatic, and in fact teems with actuality. Calmette's results in obtaining pulmonary anthracosis by mean of feeding with Indian ink, etc., are met with the simple assertion that untreated "controls" show the same condition. The author, it is clear, does not regard experiment as a fetish. The Scotch work as to the alleged excessive occurrence of bovine tuberculosis in Edinburgh is effectively dealt with also. The book is very well printed, considering the times; the author's preface not short, but good; the editors', short but pleonastic.

**Elements of Hygiene and Public Health.** By CHARLES PORTER, M.D., B.Sc., M.R.C.P. (Edin.), of the Middle Temple, Barrister-at-Law; Medical Officer of Health, Metropolitan Borough of St. Marylebone. (London: Henry Frowde and Hodder and Stoughton. 1917. 12s. 6d. net.)

DR. CHARLES PORTER'S book, although he describes it as a textbook for students and practitioners of medicine, is a "handbook" rather than a textbook. It is not, and it does not profess to be, a textbook of hygiene for candidates for a diploma in State medicine. It is intended, and is admirably fitted, to supply the medical man with a general survey of the work of the medical officer of health in his relationship to the general practitioner. It is devoid of formulæ and statistical detail. It is eminently brief in description, and is in a sufficient degree simple in matter and language to appeal to a large circle of non-medical readers—to the sanitary inspector, the health visitor, and the infant-welfare worker, and it should find a place upon the library shelves of every nurse-training school. A fairly full index makes it a work of ready reference.

That, within the 384 octavo pages of such a book, the treatment of such subjects as meteorology, tropical diseases, and vital statistics should be fragmentary and introductory is only to be expected; room is, however, found to give an outline of them, as also brief references to public health administration and school hygiene. Forty-five pages are devoted to the removal and disposal of refuse and waste, including sewage, and within this section are thirty-seven of the ninety-eight illustrations which the book contains. The illustrations generally are from fresh drawings, "the fairly common practice of showing pictures appearing in other publications" having been avoided; although we would suggest that it is unnecessary to give a figure of so familiar an article as a dummy teat, the illustrations are upon the whole good and their subjects unconventional and well chosen. One or two carelessly worded passages in the book will no doubt be revised in later editions. The vague statement that "a quantity of cream should always, if possible, be added to the feeds of a bottle-fed baby" is amplified in the model infant-feeding leaflet in the appendix. The objection to the attempt to use permanganate of potassium as a means of domestic purification of drinking-water is not only that "few persons really care to risk adding such materials to the water"; its inefficacy makes it a delusion and a snare. We believe that electric-lamp filaments are never made of "platinum." In connection with occupational ear affections it cannot be accepted that "munition workers" commonly suffer from "the sharp, penetrating reports of smaller guns"!

Dr. Charles Porter has found room for a mass of useful material, and his book will, we trust, meet with the wide demand and the high estimation which it well deserves.

**Painless Childbirth.** By Marguerite Tracy and Mary Boyd. (London: W. Heinemann. 1917. Price 7s. 6d. net.)

This is a book by two American women which repeats in substance the errors and the prejudices of the book on "Twilight Sleep" published two or three years ago by Hannah Rion. If a shade less hysterical in tone than the latter book, it is equally inspired by hatred and contempt of the medical profession, and gives an equally garbled and untrustworthy account of the present attitude of medical opinion towards the use of chloroform, ether, scopolamine, morphine, and other anæsthetics in childbirth. Like Mrs. Rion, these authors seek to bring conviction to their readers by reproducing photographs of children who were born to mothers under the influence of scopolamine-morphine. It would be as valid to publish the photographs of Cæsarean babies as proof that all children should be similarly delivered. The whole book is a tissue of muddled reasoning, misstatements, and errors of the grossest description. It is calculated to do a great deal of harm should it ever attain any wide sale amongst the public.

**How to Become a Dispenser: the New Profession for Women.** By Emily L. B. Forster, Member of the Society of Chemical Industry, late Lecturer to the Westminster College of Pharmacy. (London: T. Fisher Unwin, Ltd. Pp. 99. Price 2s. 6d. net.)

One of the more recent professions to be opened to women is dispensing, and the success which those who have entered it have achieved has created no small demand for women dispensers; and many public institutions have availed themselves of their services. The work of a dispenser requires care and skill rather than strength, and therefore it is work especially suitable for women. This little book should serve as a useful guide to those who wish to enter the dispensing profession. It describes the different qualifications which may be obtained, the training that is needed, and it discusses the openings which may be expected to be available for the qualified woman dispenser. The war has increased the demand for women dispensers as so many men have been called up, and although for the higher qualifications a prolonged training is necessary, it is possible for the student to obtain the Apothecaries' Hall "Assistants' Certificate" after only some six months' work and without the passing of any examination in general knowledge, and this opens the profession to many women who cannot spare the time and money required for the higher qualifications. A great responsibility, however, rests on the dispenser, for the life of the patients depends on her care, and therefore dispensing, like all other responsible work, should not be lightly undertaken.

**The Medical Annual: a Year-Book of Treatment and Practitioner's Index, 1917.** (Bristol: John Wright and Sons, Ltd. London: Simpkin, Marshall, Hamilton, Kent and Co., Ltd. Pp. 668. Price 10s. net.)

This is the thirty-fifth annual volume of this ever-useful publication, and in every way it fully reaches the high level which the medical profession has learned to expect from it. The production of a work such as this, so authoritative and so well provided with illustrations, would not be possible were it not that the book has a very large circulation. It contains numberless articles on all the medical discoveries and inventions of the past year, and these accounts have been written by those physicians and surgeons who have special knowledge of the subjects with which they deal. It is natural that a large portion of the book should be devoted to the medical and surgical aspect of the war, especially in connection with the surgery of wounds of the abdomen, the spinal cord, and the jaw, but no department of medicine or surgery is neglected. A very useful section of the book is that dealing with public health, with subsections on forensic medicine, State medicine, industrial diseases, toxicology, and the school medical service. In this last section we find that it is considered desirable that sanitary authorities should have the power not only of cleansing verminous school children, but also of cleansing the other inmates of vermin-infected houses, for otherwise the children will immediately become reinfected. There is certainly much to be said for this extension of the powers of the sanitary authorities, but it is not difficult to see that such powers might run the risk of being sometimes abused. There is an interesting chapter on recent inventions, and on new drugs and pharmaceutical products; and, lastly, there is a list of the chief medical works and new editions published during the past year. We think much credit is due to the editor and publishers of this work, considering the difficulties they must have encountered in obtaining contributions at a time when the medical profession is overworked.

**Poems of Purpose.** By Ella Wheeler Wilcox. (London: Gay and Hancock, Ltd. Price 1s. 3d.)

Mrs. Wilcox, like Martin Tupper, offers to criticism the curious question why "the most widely read poet of the day" should be destitute of music, originality, and beauty. Of a new poet we expect novelty of form, and originality of substance. Mrs. Wilcox's admirers would not claim that she has added anything to poetic form. Her volumes are unanimous in declaring that she has no new vision to give us. What is there then in her amiable commonplaces which makes her books run from edition to edition? What is the reason for this popularity? The question itself is the answer. Popularity is not based on reason. It is the manifestation of a mood, and it is among the mass that commonplaces have their currency. Mrs. Wilcox has only to say "There are so many little things to make life beautiful" for the public to purchase her books by the score. What other poetry, one wonders, do her admirers read? We suspect no other, for those who were ravished with rag-time were not acquainted with the syncopated rhythms of Beethoven's third "Leonora" overture. The measure of her popularity is the measure of the public's ignorance of other poets; and anyone who turns the pages of the smaller Harmsworth papers, the papers which really pay, the women's papers, will find in prose the sentiments which Mrs. Wilcox sets to rhyme so industriously. The use of such big words as "ideals," "chivalry," "high principles," without any indication of their meaning, is always popular, and Mrs. Wilcox knows how to run them into rhyme. The process is much liked, for a definition is a challenge, but repetition soothes the feelings by sending the imagination to sleep.

**The Patriot's Birthday Book.** Compiled by C. E. Thomas. (London: T. Murby and Co. 1916. Price 1s. net.)

The publishers of this book announce that they consider it well suited "for the preservation by nurses of autographs of military patients." We are not quite sure that such a practice, if it exists, is one to be much encouraged; and we believe that the title of this particular birthday book, and the extracts from the speeches of various politicians which adorn it, will be equally uncongenial to anyone who really cares for Britain and the Allied cause.

